# Bone-Immune Cell Crosstalk: Bone Diseases

**DOI:** 10.1155/2015/108451

**Published:** 2015-04-27

**Authors:** Giorgio Mori, Patrizia D'Amelio, Roberta Faccio, Giacomina Brunetti

**Affiliations:** ^1^Department of Clinical and Experimental Medicine, University of Foggia, 71100 Foggia, Italy; ^2^Department of Medical Science, Section of Gerontology and Bone Metabolism Diseases, University of Torino, 10126 Torino, Italy; ^3^Department of Orthopedics, Washington University School of Medicine, St. Louis, MO 63110, USA; ^4^Department of Basic Medical Sciences, Neurosciences and Sense Organs, Section of Human Anatomy and Histology, University of Bari, 70124 Bari, Italy

## Abstract

Bone diseases are associated with great morbidity; thus, the understanding of the mechanisms leading to their development represents a great challenge to improve bone health. Recent reports suggest that a large number of molecules produced by immune cells affect bone cell activity. However, the mechanisms are incompletely understood. This review aims to shed new lights into the mechanisms of bone diseases involving immune cells. In particular, we focused our attention on the major pathogenic mechanism underlying periodontal disease, psoriatic arthritis, postmenopausal osteoporosis, glucocorticoid-induced osteoporosis, metastatic solid tumors, and multiple myeloma.

## 1. Introduction

Bone is an active tissue that undergoes continuous remodelling by two distinct processes, bone formation and bone resorption [1]. These events are strongly linked and tightly regulated to maintain skeletal homeostasis [2]. The bone cells responsible for the dual processes include the bone resorbing cells, that is, the osteoclasts (OCs), which are differentiated cells derived from hematopoietic cells of the monocyte-macrophage lineage and bone forming cells, that is, the osteoblasts (OBs), which are of mesenchymal origin. Alteration of the differentiation/activity of OCs as well as OBs leads to bone diseases. The close relationship between the bone and the immune system has been increasingly recognized, in particular during pathological conditions in which activation of both systems occurs [3]. It is known that inflammation increase leads to an augment in the immune function, which culminates in an increased production of tumour necrosis factor (TNF) or receptor activator of NF-kB ligand (RANKL) by activated T cells, that has been linked to bone loss associated diseases (inflammatory and autoimmune disease, postmenopausal osteoporosis). Different studies have been performed to identify the T cell subset involved in osteoclastogenesis. In general, T cells could be classified as effector-cytotoxic T population (CD8+ cells) and helper T cells (CD4+ cells). CD4+ T cells, upon activation and expansion, develop into diverse T helper (Th) cell subsets secreting signature cytokine profiles and mediating distinct effector functions [4]. Until recently, T cells were divided into Th1 or Th2 cells, depending on the cytokines they produced (with Th1 producing IFN-gamma and IL-2 and Th2 producing primarily IL-4/IL-5/IL-10). Regulatory T cells (Tregs, CD4+CD25+Foxp3+) potently inhibit the function of effector T cells [4]. A third subset of IL-17-producing effector T helper cells, called Th17 cells, has been more recently discovered and characterized. Th17 cells produce IL-17, IL-17F, and IL-22, thereby inducing a massive tissue reaction owing to the broad distribution of the IL-17 and IL-22 receptors. Th17 cells support OC formation mostly through the expression of IL-17, which is recognized to induce RANK expression on OC precursors as well as RANKL production by cells supporting OC formation [4, 5]. IL-17 also makes possible local inflammation through the recruitment and the activation of immune cells, leading to the release of proinflammatory molecules, as IL-1 and TNF*α* [4]. These proinflammatory molecules increase RANKL expression and synergize with RANKL signalling to maximize OC formation. A relatively high expression of RANKL on Th17 cells may also participate in the enhanced osteoclastogenesis. Collectively, Th17 cells can be considered an osteoclastogenic Th subset; however, they are not the only ones. In fact, activated T cells, expressing high RANKL levels, have the ability to directly induce OC differentiation by acting on OC precursor cells [6].

However, because T cells/immune cells also secrete a variety of cytokines and express membrane-bound factors other than RANKL, which could support OC formation, mainly in pathological condition; this issue might be further explored, together with the mechanisms that could modulate their expression.

We describe recent efforts highlighting the prominent role of immune system in the alteration of bone remodelling, thus favouring the development of many bone diseases, such as periodontal disease (PD), psoriatic arthritis (PsA), postmenopausal osteoporosis, glucocorticoid-induced osteoporosis (GIO), metastatic solid tumors, and multiple myeloma (MM).

*Periodontal Disease*. PD is a common complex infection of the oral cavity that specifically affects the gingiva, the periodontal ligament, and the alveolar bone. It is characterized by an inflammatory response to bacteria present in the gingival pocket [7] and may remain confined to the gingiva or may progress to extreme periodontal destruction with the loss of the alveolar bone. PD is the main cause of tooth loss among adults and is associated with important alteration in facial aesthetics and defeat of masticatory and phonetics function [8]. It is also well recognized that the presence of only pathogenic bacteria is insufficient to PD. Progression of this disease occurs due to a combination of factors, including the presence of periodontopathic bacteria, high levels of proinflammatory cytokines (IL-1, TNF*α*, IL-4, IL-6, IL-8, and IL-11), prostaglandin E2 (PGE_2_), low levels of anti-inflammatory cytokines including IL-10, transforming growth factor (TGF-*β*), and retinoic acid [9]. Genetic factors increase the susceptibility of some individuals in developing this inflammatory disease. It has been supported by reports of familial aggregation of severe forms of the disease [10], and twin studies [11]. Recent candidate gene studies for periodontal disease have focused on genes related to host immunity and inflammatory response such as cytokines, cell-surface receptors, chemokines, enzymes, and antigen recognition. Histological examination of periodontitis lesions reveals that the granulocytes appear to play a key role in the maintenance of the periodontal health. These cells are present in the junctional epithelium in large numbers and they isolate tissues from the bacteria action; thus, severe forms of periodontitis frequently affect patients with diseases such as leukocyte adhesion deficiency and neutropenia. The failure of granulocytes to transmigrate into the endothelium results in an increase on the inflammatory response and reduces the protective response against periodontal pathogens. In the presence of active disease, the epithelial migration causes a deep periodontal pocket resulting in bacterial invasion, inflammation, and destruction of the connective tissue, with subsequent bone loss and possible tooth loss. Langerhans cells and dendritic cells of bone marrow origin, that are located within the epithelium, are a connecting link with acquired immunity. The adaptive immune response is activated when the epithelial barrier, with its innate system, is penetrated. The dendritic cells participate to the innate inflammatory response and moreover they capture and present antigens to B and T cells of the acquired immune system [12]. Activated CD4 T helper cells produce subsets of cytokines with different immune responses: Th1 and Th2 cells, respectively, associated with cellular and humoral immunity [13]. The recently described Th17 and Treg cells have antagonistic roles as effector and suppressive cells [8]. B cells differentiate into plasma cells producing specific antibodies. Thus, tissues affected by periodontitis become colonized with both lymphocytes subtypes with B cells being more represented than T cells. In a not progressive lesion, IFN-*γ* increases the phagocytic activity of both neutrophils and macrophages and hence contains the infection. In case of a reduced innate immune response, a consequent weak Th1 response may not contain infection. Moreover, activated mast cells determine a Th2 response, B cell activation, and antibody production. The antibodies can control the infection or, as in the case of production of IgG2 in large amount, the lesion will persist. Sustained B cell activation may lead to IL-1 secretion and periodontal disease progression. Th17 cells have been identified in the periodontal tissues. IL-17 mainly produced by Th17 has been shown to stimulate epithelial, endothelial, and fibroblastic cells to produce IL-6, IL-8, and PGE_2_, thus sustaining the disease progression. In addition, IL-17 induces RANKL production by osteoblasts stimulating bone resorption. It has been demonstrated that* periodontitis bacteria* induce a significant increase in the production of IL-17 [14]. According to recent studies, IL-17 significantly enhances RANKL and inhibits osteoprotegerin (OPG) expression in human periodontal ligament cells [15]. It has been hypothesized that Th17 cells may be involved in Th1 modulation and enhanced inflammatory mediators' production by gingival fibroblasts in periodontal disease. Circulating T cells express high levels of RANKL and spontaneously promote osteoclastogenesis in patients [16]. Th1 and Th17 cells, as well as B cells, increase RANKL expression [17]. Other studies demonstrated that also B cells produce RANKL in response to periodontal pathogen stimulation [17]. Contrarily, Treg cells decrease RANKL secretion whereas TGF-*β* stimulates Treg cell differentiation. This process is supported by retinoic acid and counteracted by IL-6 and IL-1. In chronic inflammatory disease as PD, retinoic acid levels are suppressed and Treg activity is inhibited in favor of Th17 pathogenic effect [18]. In PD, proinflammatory cytokines overcome anti-inflammatory ones, and Th17 cells surmount Treg: this inflammatory state determines the destruction of connective tissue and alveolar bone.

*Psoriatic Arthritis*. Psoriasis is a chronic inflammatory disease of the skin; a considerable part of patients with psoriasis develops an inflammatory arthritis characterized by increased bone remodeling with osteolysis called PsA [19].

The mechanisms responsible for the development of the PsA should be better explained, but the immune system plays the main role in the pathogenesis of this disorder, that has to be considered as a chronic inflammation. Therefore, patients with psoriasis have elevated levels of circulating neutrophils. The Th cells play an important role; in particular, Th1 and Th17 are involved in the pathogenesis of the disease. PsA is characterized by T and B cell infiltrates and neoangiogenesis in the synovial membrane and by the overexpression of inflammatory cytokines. PsA synovitis is indicated by hyperplasia of the synovial lining cells and mononuclear cell infiltration. Moreover, ectopic lymphoid neogenesis appears. Fibroblasts and T cells in PsA synovial fluid induce osteoclastogenesis and bone resorption, mediated by RANKL, TNF-*α*, and IL-7 [37]. Inflammatory cytokine set such as TNF-*α*, IL-1*β*, IL-10, IFN-*γ*, IL-12, IL-15, IL-17, and IL-18 were highly expressed in synovial fluid of PsA patients, while fibroblasts isolated from their skin and joints secreted IL-1 and IL-6; some of these cytokines have also recognized osteoclastogenic features.

Both T cell suppression and TNF-*α* inhibitors are effective in humans in the treatment of psoriasis. In PsA patients, there is a great increase in the number of peripheral blood Th17 cells. Thus, recent studies indicated that Th17 cells [38] are the cells most significantly involved in psoriasis. Like Th1 and Th2 cells, Th17 cells appear to be evolved in inducing acquired immune responses against microorganisms, such as bacteria. Abnormal Th17 responses are believed to play a significant role in the onset of various autoimmune diseases. Moreover, IL-23 is indispensable for Th17 effector functions in immune disorders and maintenance of Th17 cells.

Orphan nuclear receptor ROR*γ*t (retinoid-related orphan receptor gamma t) has been identified as specific Th17 transcription factor [39]. ROR*γ*t is involved in the production of IL-23 receptor (IL23R) which is expressed by monocytes, Th1, Th0, Th17, NK, and dendritic cells. IL23R has an important role in stimulating Th17 cells. IL-23 receptors promote IL-17 transcription and Th17 cell differentiation via enforced ROR*γ*t expression. IL-23 acts on cells that have been differentiated into Th17 cells, potentiating ROR*γ*t activity, and participates in maintenance and proliferation of Th17 cells.

Clinical trials studying the effects of anti-IL-17 and anti-IL-23 neutralizing antibodies in PsA patients are in progress [29], while the first results seemed to be not as impressive as those for TNF-*α* inhibitor therapy, a recent clinical trial indicated that Brodalumab, an IL-17RA inhibitor, determined a significant improvement, when administered for 12 weeks to PsA patients [36]. Moreover, new studies demonstrated that Ustekinumab, a monoclonal antibody against both IL-12 and IL-23 cytokines, interfering, respectively, with Th1 and Th17 activity, improved significantly PsA symptoms, although similar efficacy of TNF-*α* inhibitors needs about 52 weeks of treatment to be achieved [33]. Although immune responses mediated by IL-17 and IL-23 are not as evident as those with TNF-*α*, Th17 cells appear to play an important role in PsA.

The activation of natural immunity in PsA stimulates Th17 and Th1 cells, which sustain autoimmune pathology. There is an interesting report regarding the relationship of PsA with microbial infection that is suggestive of PsA pathogenesis [40].

The observation of the lack of symptoms improvement in PsA patients underwent to HIV infection and thus to CD4+ reduction, suggested that Th cells cooperate in the pathogenesis with CD8+ cells [41]. Probably, CD8+T cells potentiates the production of cytokines in the synovial membrane, and the cytokines induce fibroblast proliferation promoting fibrosis [42–44], that probably contribute to joint stiffness and ankylosis [45].

*Postmenopausal Osteoporosis*. Postmenopausal osteoporosis is a systemic skeletal disorder characterized by reduced bone mineral density and microarchitectural deterioration of bone tissue resulting in fragility and susceptibility to fractures [46] and uncoupling of osteoblast-mediated bone formation and osteoclast-mediated bone resorption. Postmenopausal osteoporosis stems from the cessation of ovarian function at menopause and from genetic and nongenetic factors which heighten and prolong the rapid phase of bone loss characteristic of the early postmenopausal period. OC activity increases after menopause; these cells may be considered as cells at the crossroad between immune system and bone as their precursors circulate within the mononuclear fraction of peripheral blood [47–53] and they interact with other immune cells as T cells [54].

OC precursors increase during estrogen deficiency [47] and in condition characterized by increased bone turnover as bone metastases [50, 55] or inflammatory diseases [55–57].

Estrogens act on OC formation and activity both directly and indirectly, in particular their action is mediated through the influence on immune system [54]. In particular, estrogen loss upregulates OC formation and activity through an increased production of proosteoclastogenic cytokines by bone marrow cells [58], OBs [59], and immune cells [47, 60].

Proinflammatory and proosteoclastogenic cytokines as macrophage colony stimulating factor (M-CSF) and RANKL are increased during estrogen deficiency [61, 62].

Additional inflammatory cytokines are responsible for the upregulation of OC formation observed during estrogen deficiency; some of these molecules have a well-established role in osteoclastogenesis and bone loss, while others have not. Among these molecules, the most involved ones in estrogen deficiency bone loss appear to be TNF-*α*, IL-1, IL-7, and IL-17 [1, 63–70]. A key role of T cell-produced TNF*α* has been demonstrated also in bone metastasis [51, 71].

Estrogens are key regulators of immune function as demonstrated both in animals and in humans [72, 73]. Despite some inverse reports [74, 75], the main body of literature firmly supports the essential role of activated T cells in regulating bone loss induced by estrogen deficiency [47, 69, 72, 76–79].

In humans, we have demonstrated a fundamental role for T cells in postmenopausal bone loss. In particular, we showed that osteoclastogenesis from peripheral blood precursors occurs only in the presence of T cells and that T cells are more active than in healthy post- and premenopausal controls [47]. T cells from osteoporotic patients produce more RANKL and TNF-*α*, thus inducing OC formation and activity [47]. It has also been demonstrated that hormone replacement therapy decreases osteoclastogenic cytokine production in postmenopausal women. RANKL expression on lymphocytes and marrow stromal cells is significantly elevated during estrogen deficiency in humans and correlates directly with increases in bone resorption markers and inversely with serum estrogen levels [77].

Estrogen loss promotes T cell activation by increasing antigen presentation [76, 80] and increases thymus output of T cells into peripheral blood [68]. Estrogen loss expands the proliferation and lifespan of bone marrow T cells [76, 78] increasing expression of class II transactivator (CIITA), a transcriptional coactivator acting on MHCII promoter [76, 81, 82].

Estrogen deficiency increases the number of activated CD40L-expressing T cells that promote the expression of M-CSF and RANKL by stromal cells and downregulates the production of OPG. The net result is a significant increase in the rate of osteoclastogenesis [83, 84]. This mechanism was also described in bone loss due to increased PTH levels [85, 86]. It is known that the CD40/CD40L system is crucial for T cell activation and several functions of the immune system. It promotes macrophage activation and differentiation, antibody isotype switching, and the adequate organization of immunological memory in B cells.

Also, the Th17 cells have been implicated in ovariectomy-induced bone loss; these cells increased after ovariectomy and stimulate osteoclastogenesis through IL-17 production [69]. This effect is reversed by treatment with estradiol. IL-17 increases OB production of proosteoclastogenic cytokines as TNF*α*, IL-6, and RANKL; these effects are antagonized by estradiol.

Activated T cells have also been suggested to inhibit osteoclastogenesis by diverting early OC precursors towards dendritic cells differentiation [87]. Indeed T cells have the capacity to generate both osteoclastogenic cytokines such as RANKL and TNF-*α* [47], as well as antiosteoclastogenic factors such as IL-4. It has also been suggested that the effects of activated T cells on osteoclastogenesis* in vitro* depend on the manner in which they are activated [88]. The net effect of T cells on OC formation may consequently represent the prevailing balance of anti- and proosteoclastogenic T cell cytokine secretion. However, in humans, T cells seem to be proosteoclastogenic in different diseases including estrogen deficiency [47, 56, 66, 89–91].

Taken together, these observations demonstrate the causal relation among estrogen deprivation, T cell activation, increased cytokines production, and bone demineralization.

Also another type of immune cell the B cell has recently been studied as directly implicated in the regulation of bone resorption and may be directly involved in the pathogenesis of postmenopausal osteoporosis. Recent data have shown that B cells are the dominant producers of OPG in the bone microenvironment* in vivo* [79]. In fact, B cell KO mice have an osteoporotic phenotype with enhanced osteoclastic bone resorption and reconstitution with B cells by adoptive transfer, completely rescued mice from development of osteoporosis, and normalizing OPG production [79].

In human and animal B cells, OPG production can be significantly upregulated by the activation of CD40 [79]. In line with these data, both CD40 and CD40L KO mice displayed an osteoporotic phenotype and a significant deficiency in bone marrow OPG concentrations [79].

Thus, the emerging data suggest that the B lineage, rather than the OB lineage, is likely the major source of OPG in the bone microenvironment and that T cell signalling to B cells, through the costimulatory molecules CD40L and CD40, plays an important role in regulating basal OC formation and in regulating bone homeostasis.

On the other hand, it has been recently demonstrated that activated B cells overexpress RANKL, contributing to bone resorption [92, 93] and that ovariectomy in mice increases the number of RANKL-expressing B lymphocytes in the bone marrow [94].

A recent paper shows that mice lacking RANKL in B cells were partially protected from the ovariectomy-induced loss of cancellous bone [60]. The role of B-lymphocytes has also been evaluated in disease characterized by focal bone loss as in periodontal inflammation [66, 69, 95] and rheumatoid arthritis [93]. In rheumatoid arthritis, a recent paper suggests that B cells depletion ameliorates the suppressed bone turnover [96].

Taken together, these data suggest that B-lymphocyte involvement in the adaptive immune response contributes to bone resorption by the upregulation of RANKL expression through Toll-like receptor pathways and aligns with the known ability of T cells to produce RANKL in the presence of immune stimulus and to increase osteoclastogenesis. The effect of estrogen deficiency on B cell modulation may be one of the mechanisms through which menopause affects bone metabolism.

Thus, the involvement of T and B cells in the control of bone turnover may provide a novel explanation for the propensity to osteopenia and osteoporosis development after the cessation of ovarian function.

*Glucocorticoid-Induced Osteoporosis*. GIO is the most frequent origin of secondary osteoporosis in adults due to the direct effects of glucocorticoids (GCs) on bone cells [97]. GCs primarily affect trabecular bone, whereas the cortical bone mass is reduced to a lower and slower extent. Thus, fractures of the vertebrae are more recurrent. GC exposure determines a rapid and early phase of bone loss, which is the consequence of bone resorption exacerbation. This phase is followed by a more chronic and progressive phase in which bone mass declines because of impaired OB activity. GCs augment RANKL expression and reduce OPG levels in stromal and osteoblastic cells leading to the initial phase of rapid bone loss. Further, GCs increase MCSF expression as well as receptor subunits for osteoclastogenic cytokines of the gp130 family. However, the main pathophysiological mechanism of GIO is the impaired bone formation, due to reduced OB formation and activity [97, 98]. GCs impair on OBs the synthesis of type I collagen, the major protein in bone matrix. GCs may also influence osteocyte metabolism and function, modifying the elastic modulus adjacent to the osteocyte lacunae leading to reduced mineral to matrix ratios in the same areas with an enlargement of the lacunar size. Besides the GC direct actions on bone cells, GC extraskeletal effects on calcium metabolism have been reported. In particular, GCs decrease renal tubular calcium reabsorption and calcium absorption from the gastrointestinal tract is reduced by mechanisms that oppose vitamin D action [97].

GCs also impair bone metabolism during the growth. In particular, in animal models, GC administration during growth is the cause of decreased bone formation and resorption, reductions in the age-dependent increases in trabecular bone mineral and trabecular thickness, and reductions in linear growth and accrual of cortical thickness in the femur [99].

A decrease of bone mineral density (BMD) has been reported in numerous pediatric diseases that require GCs, both as long term replacement therapy, such as 21-hydroxylase deficiency (21-OHD), and as treatment of acute phase, such as asthma, systemic lupus erythematosus, juvenile rheumatoid arthritis, inflammatory bowel disease, organ transplantation, and steroid sensitive nephrotic syndrome [22]. In particular, in 21-OHD patients on chronic GC therapy, the high osteoclastogenic potential of peripheral blood mononuclear cells has been reported [100]. It is supported by both the presence of circulating OC precursors and RANKL released by T cells [100]. Further, high dickkopf-1 (DKK1) levels, a secreted antagonist of the Wnt/*β*-catenin pathway, have been demonstrated in sera and circulating monocytes, T cells, and neutrophils from 21-OHD patients [28].

*Multiple Myeloma*. MM is a haematological malignancies, characterized by the clonal proliferation of plasma cells in the bone marrow [101]. A major number of mechanisms have been proposed to explain the increased formation and activity of the bone resorbing cells, the OCs in MM bone disease, whereas few mechanisms have been identified to explain the impairment of the bone forming cells, the OBs. In particular, MM cells produce different cytokines that directly or indirectly affect the bone cell activity, such as IL-6, MIP-1alpha, IL-3, DKK1, and sclerostin [101–103]. The proposed mechanism is that MM cells adhere to bone marrow stromal cells (BMSCs) and induce the secretion of numerous proosteoclastogenic and antiosteoblastogenic cytokines. The adhesion involved integrins such as CTLA4-1 and VLA-4 expressed by MM cells and VCAM-1 expressed on BMSCs [101].

Moreover, it has previously demonstrated an important role of T cells in supporting the formation and survival of OCs from peripheral blood mononuclear cells (PBMCs) isolated from MM patients with osteolysis, through the expression of high levels of RANKL and decoy receptor 3 (DcR3) [104, 105]. Interestingly, Giuliani et al. showed that malignant human myeloma cells stimulate RANKL expression in T cells [66]. Additionally, other authors demonstrated the high expression levels of IL-17 in T cells from MM patients [30–32]. IL-17 plays a key function in the progression of bone disease in MM, since the levels of IL-17 are higher in the more advanced bone disease. IL-17 is also able to increase RANKL expression on BMSCs, thus determining osteoclastogenesis increase and consequently the development of bone lesions [31]. The amount of Th17 in the bone marrow positively correlated with the number of osteolytic lesions [31] as well as the clinical tumor stage [106]. Very recently, the involvement of LIGHT/TNFSF14 has been reported in MM-bone disease [21]. LIGHT is a newly identified member of the TNF superfamily, expressed by activated leukocytes [21]. Recent literature data linked the high serum levels of LIGHT with the bone loss associated with rheumatoid arthritis [107]. Higher expression levels of LIGHT were found in CD8+ T cells, monocytes, and neutrophils from osteolytic MM patients with respect to the same cells from asymptomatic MM patients as well as monogammopaty of undetermined significant (MGUS) and healthy subjects. Further, LIGHT inhibition significantly reduces OC formation from PBMCs of osteolytic MM patients and stimulates OB differentiation in cultures derived from MM bone marrow mononuclear cells, as demonstrated by the increase of colony forming units of OBs and by the upregulation of osterix transcription factor, bone sialoprotein, and osteocalcin bone matrix proteins.

*Bone Metastatic Tumors*. The skeleton is the predominant metastatic site for many cancers, including breast, prostate, and lung cancers [108, 109]. Tumor invasion into bone is associated with dramatic skeletal related events (SRE) such as fractures, bone pain, hypercalcemia, and spinal cord compression [110]. The current model for the pathophysiology of bone metastasis centers on the interaction between tumor cells and OCs and is known as the “tumor/bone bone vicious cycle.” Tumor cells secrete a plethora of factors and cytokines that can directly stimulate OC activation. Once mature OCs start to resorb the bone, they release bone-stored factors, such as TGF-*β*, that further stimulate tumor cell recruitment and proliferation [111]. Animal studies have shown that antiresorptive therapies protect from SRE and reduce tumor burden. Thus, antiresorptive agents, such as zoledronic acid (ZOL) and the anti-RANKL monoclonal antibody (Ab), denosumab, are widely used in the clinic in patients with bone metastasis [23, 24, 108]. Despite reducing tumor-associated bone complications, recent meta-analysis studies show controversial results on the antitumor effects of OC blockade in breast cancer patients with bone metastasis [112, 113]. A significant fraction of breast cancer patients with bone metastases shows progression in their bone disease while they are on potent antiresorptive agent treatment [114–116]. A recent study suggested the existence of a preosteolytic early phase of bone metastasis that is independent of OC activation [117]. Considering the complexity of the bone microenvironment, serving as home to hematopoietic stem cells and their progeny, which constitute the immune system, it is logical to consider the interactions between tumor cells and immune cells as potentially important regulators of bone metastasis beyond the OC.

Presence of activated CD4+ and CD8+ T cells has been observed in the bone marrow of untreated patients with breast cancer [118]. CD8+ T cells have the capacity to specifically identify and eliminate tumor cells via recognition of tumor-specific antigens. Activated CD4+ T cells can further facilitate the development of cytotoxic CD8+ T cells by secreting numerous cytokines, including Interferon *γ* (IFN). Interferon *γ* (IFN) exerts antiproliferative [119], proapoptotic [120], and angiostatic [121] effects resulting in the killing of a proportion of the tumor. Thus, presence of CD4+ and CD8+ T cells at tumor site is a good prognostic indicator. However, whether T cells modulate bone metastatic dissemination and/or tumor growth in the bone microenvironment is not totally clear. In a recent report, Bidwell et al. demonstrated that silencing of IFN regulatory factor (Irf)7, a transcription factor controlling the induction of IFN genes, in breast cancer cells promotes bone metastases through escaping from immune control [122]. Importantly, an association with low expression of Irf7 signatures in primary breast tumors and higher number of bone metastatic events has been observed [122]. This finding is a strong indication that the immune system can modulate metastatic dissemination to bone in breast cancer patients.

Using animal models with established T cell immune deficiencies, we have also demonstrated that CD4+ and CD8+ T cell populations exert antitumor effects in the context of bone metastases [123]. We found that depletion of both CD4+ or CD8+ T cell subsets can reduce the antitumor effects ZOL in animals with bone metastases. Importantly, ZOL treatment is still highly effective in suppressing tumor-induced bone loss [123]. Conversely, T cell activation induced by administration of anti-CTLA4 Ab can significantly reduce bone tumor burden [123]. These observations have important clinical implications and suggest that reduced T cell numbers or impaired T cell activation might be the cause for the failure of ZOL to reduce tumor burden and increase survival in breast cancer patients.

Developing neoplasms can also acquire the ability to escape CD8+ T cell cytotoxicity by promoting expansion of Th2-polarized CD4+ T helper and regulatory T cells, as well as immune suppressor cells of myeloid origin reviewed in [124–126]. Monteiro et al. recently found that CD4+ T cells isolated from bone marrow of tumor bearing mice are potent stimulators of osteoclastogenesis [126]. This subset of tumor-specific CD4+ T cells has the ability to promote OC activation and induce osteolytic bone disease even before seeding of tumor cells in the bone microenvironment. Importantly, when tumor-specific CD4+ T cells are adoptively transferred into mice orthotopically injected with 4T1 tumor cells, tumor colonization to bone, but not to other metastatic sites, is increased. Whether this particular population of CD4+ T cells is increasing tumor bone metastases by affecting the OCs or also by inducing an immune suppressive environment needs to be established.

The bone microenvironment is particularly enriched in a highly heterogeneous population of immature myeloid progenitor cells that have the ability to exert immune suppressive effects in the presence of a tumor. This immature myeloid population, herein referred to as myeloid derived suppressor cells (MDSCs), represents 30–40% of the total bone marrow cells of naïve mice and is further expanded up to 60–70% of total marrow cells depending on the tumor type [127]. Circulating MDSCs are detected in the blood of patients with various types of cancer [128]. In response to factors secreted by a tumor, MDSCs leave the bone marrow and are found in high numbers in circulation, spleen, and tumor sites where they induce suppression of cytotoxic T cells [129]. MDSCs exert their proneoplastic effects through the release of small soluble oxidizers, by altering T cell/antigen recognition, and depletion of essential amino acids from the local extracellular environment, all ultimately leading to T cell suppression [130–133]. In addition, MDSCs can induce the expansion of regulatory T cells, a subtype of T cells exerting immune suppressive functions. Furthermore, direct effects of MDSCs on tumor proliferation through overproduction of cytokines and angiogenic factors have also been proposed [134].

A correlation between high MDSC numbers, advanced stage of malignancy, and poor prognosis has been observed. We have recently shown that increased bone metastasis in PLC*γ*2−/− mice is due to suppression of antitumor T cells responses. Although PLC*γ*2 is not expressed by T cells, we found that PLC*γ*2−/− mice have increased MDSC numbers with more potent immune suppressive effects than WT [135]. Downregulation of PLC*γ*2 activation also occurs in the MDSCs of patients with advanced pancreatic cancer [135].

Recent evidence also indicates that MDSCs participate in the preparation of premetastatic niches where they create a favorable environment for subsequent tumor colonization [136, 137]. Accumulation of MDSCs in bone marrow has been observed during early stages of MM [138]. A role for MDSCs in promoting tumor growth in bone through the OCs has also been proposed. Zhuang et al. discovered that MDSCs from mice injected with MM cells have increased osteoclastogenic potential. Importantly, coinjection of tumor-challenged MDSCs together with MM cells leads to increased tumor burden and osteolytic lesions, an effect that is inhibited by administration of ZOL [139]. Similarly, Sawant et al., using an immune competent model of breast cancer bone metastases, showed that MDSCs isolated from the tumor bone microenvironment differentiate into resorbing OCs* in vitro*. Remarkably, MDSCs isolated from tumor-free mice or tumor-bearing animals without bone metastases lack the ability to undergo OC differentiation [140]. This important observation suggests that there are intrinsic differences between MDSCs, depending on the tumor location. Why MDSCs from mice bearing bone metastases have the ability to differentiate into OCs might depend on the proosteoclastogenic rich cytokine milieu that characterizes the tumor bone microenvironment. However, it is unlikely that the bone tumor promoting effects of this subset of MDSCs is primarily dependent on their ability to differentiate into OCs. PLC*γ*2−/− mice display increased bone metastatic dissemination and higher MDSC numbers, but deletion of PLC*γ*2−/− also impairs the OC differentiation process [123, 135]. Thus, in the context of PLC*γ*2−/− deficiency, MDSCs are more likely to support tumor growth in bone by suppressing T cell activity. All together, these studies indicate that MDSCs are central players in the tumor/bone vicious cycle either through suppression of antitumor T cell responses or through differentiation into resorbing OCs.

Unfortunately to date there is no curative treatment for bone metastasis. Tumor cells that reach the bone environment are usually resistant to the current antitumor therapeutic approaches. The only options for these patients are palliative treatments to reduce bone pain and prevent additional bone destruction. More studies are needed to exploit the importance of antitumor and tumor promoting immune responses in patients with bone metastases and whether manipulation of T cell-MDSC interactions could offer therapeutic advantages to maximize the antitumor effects of OC blockade.

## 2. Conclusions

The reviewed mechanisms underlying the bone disease clearly highlighted the key involvement of the cells with an immunological role. Further, it is also clear that numerous pathways are common to the different diseases, whereas others are disease-specific. Thus, these recent findings represent an important issue, leading to the identification of new therapeutic targets, mainly biological drugs, which in the last years are in strong development (Table 1).

## Figures and Tables

**(a):** 

Established therapeutic targets	Pathologies	References
TNF-*α*	PsA	[20]
RANKL	PsA, osteoporosis, MM, and bone metastatic tumors	[22–27]
IL-17	PsA	[29]
IL-23	PsA	[29, 33]
IL-17RA	PsA	[36]

**(b):** 

Possible novel therapeutic targets	Pathologies	References
LIGHT	MM	[21]
DKK1	Bone metastasis and GIO	[28]
IL-17	MM	[30–32]
MDSC targeting	Bone metastasis	[34, 35]
